# Tri-ponderal mass index in survivors of childhood brain tumors: A cross-sectional study

**DOI:** 10.1038/s41598-018-34602-5

**Published:** 2018-11-05

**Authors:** E. Danielle Sims, Kuan-Wen Wang, Adam Fleming, Donna L. Johnston, Shayna M. Zelcer, Shahrad Rod Rassekh, Sarah Burrow, Lehana Thabane, M. Constantine Samaan

**Affiliations:** 1 Department of Pediatrics, McMaster University, Hamilton, Ontario Canada; 2 Division of Pediatric Endocrinology, McMaster Children’s Hospital, Hamilton, Ontario Canada; 3 Division of Pediatric Hematology/Oncology, McMaster Children’s Hospital, Hamilton, Ontario Canada; 4 Division of Pediatric Hematology/Oncology, Children’s Hospital of Eastern Ontario, Ottawa, Ontario Canada; 5 Pediatric Hematology Oncology, Children’s Hospital, London Health Sciences Center, London, Ontario Canada; 6 Division of Pediatric Hematology/Oncology/BMT, Department of Pediatrics, British Columbia’s Children’s Hospital, Vancouver, BC Canada; 7 Division of Orthopedic Surgery, Department of Surgery, McMaster University Medical Centre, Hamilton, Ontario Canada; 8 Department of Health Research Methods, Evidence and Impact, McMaster University, Hamilton, Ontario Canada; 9 Department of Anesthesia, McMaster University, Hamilton, Ontario Canada; 10 Centre for Evaluation of Medicines, St. Joseph’s Health Care, Hamilton, Ontario Canada; 11 Biostatistics Unit, St Joseph’s Healthcare-Hamilton, Hamilton, Ontario Canada

## Abstract

Survivors of childhood brain tumors (SCBT) face a higher risk of cardiometabolic disorders and premature mortality compared to the general population. Excess adiposity is a known risk factor for these comorbidities. However, while SCBT have higher adiposity compared to healthy controls, measuring adiposity in clinical practice involves access to specialized equipment and may impact busy clinical services. Tri-ponderal Mass Index (TMI; kg/m^3^) may be a superior measure of adiposity when compared to Body Mass Index (BMI; kg/m^2^). However, its use in determining adiposity in SCBT has not been assessed. This study aims to validate TMI as a clinical measure of adiposity in SCBT. This was a cross-sectional study including 44 SCBT (n = 20 female) and 137 (n = 64 female) non-cancer control children, 5–17 years of age. BMI and TMI were calculated from height and weight measurements. Fat mass percentage was assessed using bioelectrical impedance analysis and waist to hip and waist to height ratios were used to assess central adiposity. Regression analyses were adjusted for age, sex, puberty and treatment. TMI demonstrated strong correlations to measures of total and central adiposity and predicted adiposity in SCBT and non-cancer controls, with stronger trends in the latter group. TMI may serve as a reliable clinical measure of adiposity in both SCBT and healthy children.

## Introduction

Obesity has contributed to the rise of cardiovascular diseases and type 2 diabetes, making them some of the most significant and costly healthcare challenges of the 21^st^ century^1–6^. One group that is especially impacted by these chronic diseases include childhood cancer survivors^7–9^. Within this population, survivors of childhood brain tumors (SCBT) represent an emerging group that has been recently reported to develop stroke and type 2 diabetes at higher rates than those seen in non-cancer control populations^10–12^. Obesity leads to increased cardiovascular mortality at a relatively young age in SCBT^12–22^.

Excess adiposity, especially visceral adiposity, has been linked to cardiovascular disease and type 2 diabetes in the general population, and SCBT have more adiposity compared to healthy controls^23–27^.

While adiposity is a potentially modifiable risk factor for cardiometabolic risk, measuring adiposity in clinical practice can be time consuming in the clinical setting, and requires specialized equipment including bioelectrical impedance scales or Dual-energy X-ray Absorptiometry (DXA) scans^28–30^. The availability of feasible and reliable clinical measures of adiposity will circumvent these limitations and help prioritize SCBT for closer monitoring and targeting them in interventions in an attempt to improve outcomes.

The tri-ponderal mass index, defined as weight divided by height cubed (TMI, kg/m^3^), is an alternate measure of adiposity in children^31^. TMI is reported as a more accurate predictor of adiposity compared to Body Mass Index (BMI, kg/m^2^) and BMI z-score when validated against DXA scans in adolescents^31^. An important advantage of TMI is that it uses the same height and weight measurements used to calculate BMI. Thus, TMI may offer a feasible clinical measure to assess adiposity. However, TMI has not been validated as a measure of adiposity in SCBT. Our aim is to validate TMI as a clinical measure of adiposity in SCBT and compare this group to non-cancer controls.

## Results

### Population characteristics

The characteristics of participants are reported in Table 1. We included 44 SCBT (n = 20 female, 45.50%), and 137 non-cancer controls (n = 64 female, 46.70%).

**Table 1:** Study Population Characteristics.

Variables	SCBT (n = 44)	Controls (n = 137)	p-value (between groups)
	Mean ± SD	Mean ± SD	
Age at enrollment (years)	11.16 ± 4.30	14.03 ± 2.60	<0.001
Sex, No. (%)			
Male	24 (54.60)	73 (53.30)	0.88
Female	20 (45.40)	64 (46.70)	
Puberty			
Pre-pubertal	21 (47.70)	16 (11.70)	<0.001
Pubertal	23 (52.30)	121 (88.30)	
Height (cm)	141.80 ± 25.00	162.70 ± 15.00	<0.001
Weight (kg)	43.20 ± 22.40	59.20 ± 20.60	<0.001
BMI z-score	0.57 ± 0.94	0.45 ± 1.10	0.51
BMI percentile (%)	67.60 ± 26.30	62.20 ± 29.30	0.33
TMI (kg/m^3^)	14.10 ± 2.50	13.50 ± 2.90	0.099
Fat mass percentage	24.10 ± 9.30	22.20 ± 9.40	0.16
Waist-to-hip ratio	0.87 ± 0.07	0.82 ± 0.07	<0.001
Waist-to-height ratio	0.46 ± 0.06	0.45 ± 0.07	0.17

Abbreviations: SCBT, survivors of childhood brain tumors; SD, standard deviation; BMI, body mass index; TMI, tri-ponderal mass index.

The SCBT and control groups had similar sex (p = 0.88) and ethnic distribution (Caucasian SCBT: n = 34 (77.30%); controls: n = 91 (66.40%), p = 0.18). SCBT were younger than the non-cancer controls (SCBT: 11.16 ± 4.29; controls: 14.03 ± 2.56, p < 0.001), and fewer survivors were pubertal compared to controls (SCBT: n = 23 (52.30%); controls: n = 121 (88.30%), p < 0.001). Survivors had lower weight (p < 0.001) and were shorter (p < 0.001) compared to controls.

In relation to body mass measures, we confirmed previous similar trends in both survivors and controls of BMI z-score (SCBT:0.57 ± 0.94; controls: 0.45 ± 1.12, p = 0.51) and BMI percentile (SCBT: 67.60 ± 26.30%; controls: 62.20 ± 29.30%, p = 0.33).

Adiposity levels were similar between groups including fat mass percentage (%FM; SCBT: 24.10 ± 9.30%; controls: 22.20 ± 9.40%, p = 0.16) and waist-to-height ratio (WHtR) (SCBT: 0.46 ± 0.06; controls: 0.45 ± 0.07, p = 0.17). However, waist to hip ratio (WHR) was higher in SCBT when compared to non-cancer controls (SCBT: 0.87 ± 0.07; controls: 0.82 ± 0.07, p < 0.001).

Due to the age and pubertal staging differences noted, we validated the results of the analysis of the full cohort by performing a subgroup analysis that included age- and sex-matched controls. In the latter analysis age, sex, and puberty adjusted analyses revealed identical trends to those reported for the full study cohort. The data for the matched subgroup analyses are reported in Supplementary Tables S1–S3.

### Tumor characteristics and treatments

 The details of tumor characteristics and treatment methods are reported in Table 2.

**Table 2:** Brain tumor characteristics (n = 44).

Variables	No. (%)
**Brain tumor type**	
Non-NF-1, low grade glioma	18(40.90)
PNET/Medulloblastoma	8 (18.20)
NF-1, low grade glioma	9 (20.50)
CNS germ cell tumors	3 (6.80)
Subependymal giant cell astrocytoma	2 (4.50)
Ependymoma	2 (4.50)
Meningioma	1 (2.30)
Choroid plexus papilloma	1 (2.30)
**Brain tumor location**	
Supratentorial	22 (50.00)
Infratentorial	22 (50.00)
**Brain tumor treatments**	
Surgery	29 (65.90)
Radiotherapy	13 (29.50)
Chemotherapy	20 (45.50)
No treatment	8 (18.20)

Abbreviations: CNS, Central Nervous System; PNET, Primitive Neuroectodermal Tumor; NF-1, Neurofibromatosis Type 1.

The most common tumors in survivors were low-grade gliomas (n = 29 (65.90%)). Brain tumors were equally localized to supratentorial and infratentorial regions. The treatments included surgery (n = 29 (65.90%)), radiotherapy (n = 13 (29.50%)) and chemotherapy (n = 20 (45.50%)). Eight (18.20%) participants were being managed with a wait and see approach at the time of inclusion in the study.

### The association of TMI with body mass and adiposity

To assess if TMI correlates with body mass measures and adiposity, we used Spearman’s correlation test. We conducted unadjusted and age, sex, and puberty-adjusted analyses (Table 3).

**Table 3:** Spearman’s correlation of TMI and BMI z-score with adiposity measures in SCBT and controls.

Group	Variable	BMI z-score	%FM	WHR	WHtR
**Unadjusted**					
SCBT	TMI	0.87**	0.73**	0.56**	0.69**
	BMI z-score	—	0.66**	0.55**	0.71**
Controls	TMI	0.95**	0.85**	0.38**	0.83**
	BMI z-score	—	0.80**	0.40**	0.81**
Total	TMI	0.93**	0.83**	0.45**	0.83**
	BMI z-score	—	0.78**	0.45**	0.80**
**Partial Correlations - Adjusted for age, sex and puberty**					
SCBT	TMI	0.86**	0.73**	0.44*	0.82**
	BMI z-score	—	0.68**	0.46*	0.72**
Controls	TMI	0.94**	0.88*	0.46**	0.87**
	BMI z-score	—	0.90**	0.39**	0.80**
Total	TMI	0.92**	0.85**	0.46**	0.86**
	BMI z-score	—	0.85**	0.41**	0.78**

*p-value < 0.05, **p-value < 0.001.

Abbreviations: SCBT, survivors of childhood brain tumors; BMI, body mass index; TMI, tri-ponderal mass index; %FM, percent fat mass; WHR, waist-to-hip ratio, WHtR, waist-to-height ratio.

TMI levels were similar between the groups (SCBT: 14.12 ± 2.54 kg/m^3^; controls: 13.46 ± 2.86 kg/m^3^, p = 0.10). TMI correlated with BMI z-score in both groups (Unadjusted, SCBT: ρ = 0.87; p < 0.001; controls: ρ = 0.95; p < 0.001; Adjusted, SCBT: r = 0.86; p < 0.001; controls: r = 0.94; p < 0.001).

We next assessed whether TMI correlates with measures of adiposity. TMI correlated significantly with measures of total adiposity including fat mass percentage (%FM) (Unadjusted, SCBT: ρ = 0.73; p < 0.001; controls: ρ = 0.85; p < 0.001; Adjusted, SCBT: r = 0.73; p < 0.001; controls: r = 0.88; p < 0.01). In addition, TMI correlated with measures of central adiposity, including WHR (Unadjusted, SCBT: ρ = 0.56; p < 0.001; controls: ρ = 0.38; p < 0.001; Adjusted, SCBT: r = 0.44; p < 0.01; controls: r = 0.46; p < 0.001), and WHtR correlated more strongly with TMI when compared to WHR (Unadjusted, SCBT: ρ = 0.69; p < 0.001; controls: ρ = 0.83; p < 0.001; Adjusted, SCBT: r = 0.82; p < 0.001; controls: r = 0.87; p < 0.001). There were similar trends of correlations of TMI and BMI z-score with measures of central adiposity (Table 3). Taken together, the above data indicate that TMI is a stronger predictor of total adiposity than BMI z-score and is equivalent to BMI z-score in predicting central adiposity. The correlation between TMI and BMI z-score tended to be stronger in non-cancer controls compared to SCBT.

To assess whether TMI is a predictor of body mass and adiposity in SCBT, multivariable linear regression analyses were conducted adjusting for age, sex and puberty. As radiotherapy was a significant predictor of %FM (p = 0.002) in SCBT, it was also adjusted for in the regression analyses.

We calculated unstandardized (B) and standardized (β) coefficients, and both trended in the same direction. Moving forward, we report on the latter coefficient (Table 4).

**Table 4:** Linear regression analyses of TMI in SCBT and controls adjusted for age, sex and puberty

Variable	Population	Standardized coefficient β	p-value	Model Summary	
				Adjusted R Square	SE of the Estimate
**Dependent Variable: BMI z-score**					
TMI	SCBT	0.867	<0.001	0.71	0.50
	Controls	0.935	<0.001	0.88	0.38
**Dependent Variable: %FM**					
TMI*	SCBT	0.604	<0.001	0.65	0.10
	Controls	0.819	<0.001	0.81	0.09
BMI z-score*	SCBT	0.584	<0.001	0.65	0.10
	Controls	0.836	<0.001	0.83	0.08
**Dependent Variable: Waist-to-hip ratio**					
TMI	SCBT	0.425	0.004	0.20	0.03
	Controls	0.436	<0.001	0.31	0.03
BMI z-score	SCBT	0.442	0.002	0.23	0.03
	Controls	0.370	<0.001	0.25	0.03
**Dependent Variable: Waist-to-height ratio**					
TMI	SCBT	0.793	<0.001	0.67	0.03
	Controls	0.880	<0.001	0.77	0.03
BMI z-score*	SCBT	0.657	<0.001	0.58	0.03
	Controls	0.804	<0.001	0.64	0.04

Abbreviations: SCBT, survivors of childhood brain tumors; BMI, body mass index; %FM, fat mass percentage; SE, standard error. Models were adjusted for age, sex and puberty.

*Radiotherapy emerged as a significant predictor of adiposity, therefore we included it in the analysis.

TMI was a strong predictor of BMI z-scores in both SCBT and controls, with a stronger trend in the latter (SCBT: β = 0.867; p < 0.001; controls: β = 0.935; p < 0.001). TMI was a strong predictor of total adiposity (%FM) in both SCBT and controls (SCBT: β = 0.604; p < 0.001; controls: β = 0.819; p < 0.001). While it had lower correlation with WHR, TMI was associated strongly with WHtR, and the strength of this association was higher in controls compared to SCBT (SCBT: β = 0.793; p < 0.001; controls: β = 0.880; p < 0.001).

The above threads of data indicate that TMI is a strong predictor of total adiposity and WHtR. The association between TMI and adiposity appears to be stronger in controls compared to SCBT for BMI z-score, %FM and WHtR.

## Discussion

Survivors of childhood brain tumors are facing multiple comorbidities including cardiovascular disease and type 2 diabetes, which can impact their quality of life and lifespan^13–15,27,32–36^. The identification of predictors and markers of cardiometabolic risk may offer a path to stratify those in need of close observation and early intervention. In this study, we identified TMI as one such measure. TMI was an equally strong predictor as BMI z-score, total adiposity and WHtR, the latter being a stronger predictor of cardiometabolic risk compared to WHR^37–39^. As central adiposity is associated with adverse cardiometabolic outcomes^37–40^, this is of great clinical significance, as it allows the stratification of children with higher central adiposity to a care stream with closer cardiometabolic health monitoring and early more aggressive interventions.

TMI has been validated against DXA scan-measured adiposity in the general pediatric population^31^. Our data adds to the value of TMI in the general pediatric population and in a population with chronic health needs that has not been studied previously. TMI is a promising marker of adiposity that is clinically feasible and informative. The most widely used clinical measure of body mass, BMI, is a useful population-based measure to report the presence of obesity, and is used interchangeably to report adiposity^41^. However, one of the limitations of BMI is that it may not be adequate to diagnose obesity in certain populations, including adolescents^42,43^, hence the use of BMI z-score and percentile data to assess overweight and obesity in children. In addition, BMI misclassifies muscular individuals as being overweight or obese, which does not necessarily reflect their future risk of cardiometabolic disorders^44^. Furthermore, BMI has a weaker association with cardiovascular risk when compared to measures of adiposity including waist circumference and WHtR^37,45,46^.

Children require special consideration in using BMI to classify obesity and adiposity. Ratios of weight over height to various powers of rho, *ρ*, (weight/height^*ρ*^) have been explored to account for the effects of children’s growth during puberty, and the adjustment for *ρ* is critical because incorrect values misclassify tall or physically advanced children as overweight^47,48^. A *ρ* value equal to two as used in BMI is sufficient when height is constant, however during puberty changes in height increase the *ρ* value^48^.

In pre-school children, weight over height squared is adequate for assessing adiposity^47^. Adiposity generally declines between the ages 5–7 years before it begins to rise again, the adiposity rebound phase^49^. An earlier adiposity rebound than expected is associated with an increased risk of obesity and type 2 diabetes in adults^49–51^.

As children approach the peripubertal phase of growth and development, their body composition changes with increased adiposity, especially in girls^52,53^. The value of *ρ* gradually rises from two to three; children who have undergone a growth spurt due to puberty tend to be heavier when compared to less mature children at the same height^47^. The increase in weight accompanying growth spurts results in higher BMI values, therefore greater values of *ρ* are required to offset the weight gain experienced in physically advanced children.

However, body composition changes in children become more constant as they get older and *ρ* decreases back to two^47,48^. Therefore, during puberty, TMI may be a more accurate measure of adiposity in children^47^. For this reason, the use of TMI is more relevant to assess body fat mass in children and, when evaluated in adults, TMI is less reliable compared to BMI when correlated with skinfold thickness^41^.

Measures of adiposity in children have relied on technologies that may not be readily available in the clinical setting but have demonstrated accuracy in estimating the fat mass. DXA estimates of trunk and abdominal fat have demonstrated a strong association to total abdominal fat^28–30^, while Bioelectrical impedance analysis (BIA) has been validated as a measure of adiposity against DXA^54–56^. Our results demonstrate that TMI is a strong predictor of adiposity measured using BIA, which is congruent with adiposity assessments using DXA. This is another strength of this study, as it validates TMI against BIA, a common measure of adiposity that is more easily accessible than DXA.

Our results indicate that TMI may offer a better estimate of fat mass and is a potential tool for predicting adiposity in children compared to BMI z-score. While some studies have found TMI to be an appropriate measure of adiposity in children, others have suggested BMI may still be an equivalent measure^31,57^. However, these studies often include children as young as two years of age, and weight scales over height squared in this age group that makes validating TMI as a measure of adiposity in this group a future goal of research^47,57^.

There are several strengths of our study. The inclusion of non-cancer controls for comparison to the SCBT group offers validation of this measure in the general pediatric population as well as SCBT. The description of the association of TMI with measures of central adiposity is another strength, as central adiposity is not routinely assessed in clinical practice. Furthermore, the inclusion of an age and sex matched subgroup analysis validates our findings.

A larger sample size of SCBT is required to validate these results further, and to define their associations. In addition, TMI needs to be validated as a predictor of cardiometabolic outcomes which should be part of longitudinal studies.

In conclusion, TMI represents a clinically feasible measure that uses the same variables measuring BMI but demonstrate higher correlation with adiposity.

The availability of TMI as a clinical measure of adiposity will allow the stratification of patients at risk of excess adiposity to be prioritized for targeted interventions. This is critical, as these survivors are facing cardiometabolic diseases that are important emergent determinants of outcomes.

## Methods

### Participants

Participants were consecutively recruited from McMaster Children’s Hospital (Hamilton), Ontario, Canada from November 2012 to November 2017 to partake in the Canadian Study of Determinants of Endometabolic Health in Children (CanDECIDE study)^58,59^.

Children between 5–17 years of age were included. Parental informed consent was obtained for participants less than 7 years of age, and parental consent and participant assent were obtained for those 7–15 years of age. Participants provided written informed consent if they were 16 years or older. This is a secondary analysis of the CanDECIDE cohort study data and this analysis has been approved by the Hamilton Integrated Research Ethics Board. Study procedures were performed in accordance with the relevant guidelines and legal regulations.

### Anthropometric and clinical measurements

Standardized questionnaires were used to collect data on age, sex, puberty, and ethnicity^58,59^. Medical records were consulted to verify and collect data regarding tumor type, location, sidedness and treatment modalities for SCBT.

Anthropometric measurements included weight measured to the nearest 0.1 kg using an electronic weighing scale (Seca, USA) and height using a stadiometer measured to the nearest 0.1 cm. BMI (kg/m^2^) and TMI (kg/m^3^) were determined using height and weight measurements from all participants. BMI percentile was determined from the Children’s BMI Tool for Schools^60^. BMI z-scores were determined from the Centers for Disease Control and Prevention (CDC) growth chart^61^.

Fat mass percentage (%FM) was used to determine adiposity in the participants that was measured with the Tanita body fat monitor (Tanita Corporation, Illinois, USA)^62^.

### Statistical Analysis

Statistical analyses were performed using PASW version 18 statistical package^63^. Data are presented as counts with percentages for categorical variables and means with standard deviation for continuous variables. Only participants with complete datasets were included. Box plots and visual inspection were used to identify any outliers for removal from the analysis. Normality of the data distribution was assessed using the Shapiro-Wilk test^64^. In the case that variables had non-normal distributions, data were log-transformed. Sample size was calculated using the method proposed by Norman and Streiner. We calculated that we need eight subjects per variable to detect significant differences between groups^65^.

Spearman’s correlations were used to assess the relationship between TMI, BMI z-score, %FM, WHR and WHtR. To assess this relationship adjusted for age, sex and puberty, we ran a Partial Correlations test. Multivariable linear regression analyses were performed to determine the association between TMI with BMI z-score and adiposity measures (%FM, WHR and WHtR), in SCBT and controls. The dependent variable was set as %FM, WHtR, WHR or BMI z-score and the independent variables included TMI, age, sex, puberty and treatment. Results were reported as standardized *β* coefficients and associated p-values, with statistical significance set to alpha of 0.05. A model summary, including the adjusted R Square and the Standard Error of the Estimate, were also reported.

In order to validate that age and puberty differences did not affect the results, analyses were also repeated using an age- and sex-matched control group. Control participants were matched in terms of sex distribution to SCBT participants on a one-to-one ratio and age was matched to closest value, within three years of the SCBT participants.

## Electronic supplementary material

Supplementary Tables S1-S3

## Data Availability

The data for the current study used for statistical analysis are available from the corresponding author upon reasonable justification.
