# Supplementary material for: Tri-ponderal mass index in survivors of childhood brain tumors: A cross-sectional study
Source: Sci Rep. 2018 Nov 5;8:16336. doi: 10.1038/s41598-018-34602-5 (PMC6218522; doi:10.1038/s41598-018-34602-5)
Supplement: Supplementary file 1 — Supplementary Tables S1-S3 [file 41598_2018_34602_MOESM1_ESM.docx]

Tri-ponderal mass index in survivors of childhood brain tumors: A cross-sectional study

E. Danielle Sims^1,2^, Kuan-Wen Wang^1,2^, Adam Fleming^1,3^, Donna L. Johnston^4^, Shayna M. Zelcer^5^, Shahrad Rod Rassekh^6^, Sarah Burrow^7^, Lehana Thabane^8,9,10,11^ and M. Constantine Samaan^1,2,8*^

^1^Department of Pediatrics, McMaster University, Hamilton, Ontario, Canada

^2^Division of Pediatric Endocrinology, McMaster Children’s Hospital, Hamilton, Ontario, Canada

^3^Division of Pediatric Hematology/Oncology, McMaster Children's Hospital, Hamilton, Ontario, Canada

^4^Division of Pediatric Hematology/Oncology, Children's Hospital of Eastern Ontario, Ottawa, Ontario, Canada

^5^Pediatric Hematology Oncology, Children's Hospital, London Health Sciences Center, London, Ontario, Canada

^6^Division of Pediatric Hematology/Oncology/BMT, Department of Pediatrics, British Columbia’s Children's Hospital, Vancouver, BC, Canada

^7^Division of Orthopedic Surgery, Department of Surgery, McMaster University Medical Centre, Hamilton, Ontario, Canada

^8^Department of Health Research Methods, Evidence and Impact, McMaster University, Hamilton, Ontario, Canada

^9^Department of Anesthesia, McMaster University, Hamilton, Ontario, Canada

^10^Centre for Evaluation of Medicines, St. Joseph’s Health Care, Hamilton, Ontario, Canada

^11^Biostatistics Unit, St Joseph’s Healthcare-Hamilton, Hamilton, Ontario, Canada

***Corresponding Author:** Dr. M. Constantine Samaan

Department of Pediatrics,

McMaster University,

Division of Pediatric Endocrinology,

McMaster Children's Hospital,

1280 Main Street West, 3A-57,

Hamilton, Ontario L8S 4K1

Tel: 001-905-521-2100, ext. 75926

Fax: 001-905-3087548

E-mail:samaanc@mcmaster.ca

**Supplementary Table S1.** Study Population Characteristics of Age and Sex Matched Controls

| **Variables** | **Age and sex matched Controls**  **(n = 44)** | **p-value**  **(between age and sex matched groups)** |
| --- | --- | --- |
|  | **Mean±SD** |  |
| Age at enrollment (years) | 12.30±3.40 | 0.090 |
| Sex, No. (%) | | |
| Male | 24 (54.60) | 1.00 |
| Female | 20 (45.40) |  |
| Puberty, No. (%) | | |
| Pre-pubertal | 14 (31.80) | 0.13 |
| Pubertal | 30 (68.20) |  |
| Height (cm) | 153.55±19.30 | 0.01 |
| Weight (kg) | 49.89±19.60 | 0.046 |
| BMI z-score | 0.36±1.30 | 0.37 |
| BMI percentile (%) | 57.00±34.40 | 0.07 |
| TMI (kg/m^3^) | 13.40±3.00 | 0.13 |
| Fat mass percentage (%FM) | 21.60±9.10 | 0.15 |
| Waist-to-hip ratio | 0.84±0.07 | 0.026 |
| Waist-to-height ratio | 0.45±0.08 | 0.27 |

Abbreviations: SD, standard deviation; BMI, body mass index; %FM, percent fat mass; TMI, tri-ponderal mass index

**Supplementary Table S2.** Spearman’s correlations between body mass and adiposity measurements for age and sex matched controls

| **Unadjusted** | | | | | |
| --- | --- | --- | --- | --- | --- |
| **Group** | **Variable** | **BMI**  **z-score** | **%FM** | **WHR** | **WHtR** |
| **Age and sex matched Controls** | TMI | 0.95** | 0.90** | 0.56** | 0.86** |
|  | BMI z-score | - | 0.87** | 0.49** | 0.80** |
| **Total (age and sex matched groups)** | TMI | 0.94** | 0.83** | 0.58** | 0.83** |
|  | BMI z-score | - | 0.79** | 0.54** | 0.80** |
| **Partial Correlations - Adjusted for age, sex and puberty** | | | | | |
| **Age and sex matched Controls** | TMI | 0.95** | 0.91** | 0.56** | 0.88** |
|  | BMI z-score | - | 0.92** | 0.44* | 0.79** |
| **Total (age and sex matched groups)** | TMI | 0.91** | 0.84** | 0.51** | 0.86** |
|  | BMI z-score | - | 0.82** | 0.45** | 0.77** |

*p<0.05

**p<0.001

Abbreviations: BMI, body mass index; TMI, tri-ponderal mass index; %FM, percent fat mass; WHR, waist-to-hip ratio; WHtR, waist-to-height ratio

**Supplementary Table S3.** Regression analysis of age and sex matched controls adjusted for age, sex and puberty

| Variable | Standardized coefficient β | p-value | **Model Summary** | |
| --- | --- | --- | --- | --- |
|  |  |  | **Adjusted R Square** | **SE of the Estimate** |
| **Dependent Variable: BMI z-score** | | | | |
| TMI | 0.950 | <0.001 | 0.89 | 0.43 |
| **Dependent Variable: %FM** | | | | |
| TMI | 0.888 | <0.001 | 0.84 | 0.08 |
| BMI z-score | 0.890 | <0.001 | 0.85 | 0.08 |
| **Dependent Variable: Waist-to-hip ratio** | | | | |
| TMI | 0.551 | <0.001 | 0.33 | 0.03 |
| BMI z-score | 0.433 | 0.004 | 0.21 | 0.03 |
| **Dependent Variable: Waist-to-height ratio** | | | | |
| TMI | 0.895 | <0.001 | 0.77 | 0.03 |
| BMI z-score | 0.796 | <0.001 | 0.61 | 0.05 |

Abbreviations: BMI, body mass index; %FM, fat mass percentage; SE, standard error. Models were adjusted for age, sex and puberty.
